# Antioxidant Activities of *Commiphora leptophloeos* (Mart.) J. B. Gillett) (Burseraceae) Leaf Extracts Using *In Vitro* and *In Vivo* Assays

**DOI:** 10.1155/2021/3043720

**Published:** 2021-04-24

**Authors:** Maria Lúcia da Silva Cordeiro, Ana Raquel Carneiro Ribeiro, Luciana Fentanes Moura de Melo, Lucas Felipe da Silva, Gabriel Pereira Fidelis, Larissa Marina Pereira Silva, Ricardo Basílio de Oliveira Caland, Cesar Orlando Muñoz Cadavid, Cícero Flavio Soares Aragão, Silvana Maria Zucolotto, Riva de Paula Oliveira, Deborah Yara Alves Cursino dos Santos, Hugo Alexandre Oliveira Rocha, Katia Castanho Scortecci

**Affiliations:** ^1^Laboratório de Transformação de Plantas e Análise em Microscopia (LTPAM), Departamento de Bioquímica, Universidade Federal do Rio Grande do Norte (UFRN), Natal 59078-970, Brazil; ^2^Laboratório de Biotecnologia de Polímeros Naturais (BIOPOL), Departamento de Bioquímica, Universidade Federal do Rio Grande do Norte (UFRN), Natal 59078-970, Brazil; ^3^Laboratório de Produtos Naturais e Bioativos (PNBio), Departamento de Farmácia, Universidade Federal do Rio Grande do Norte (UFRN), Natal 59010-180, Brazil; ^4^Laboratório de Genética Bioquímica (LGB), Departamento de Biologia Celular e Genética, Universidade Federal do Rio Grande do Norte (UFRN), Natal 59078-970, Brazil; ^5^Laboratório de Controle de Qualidade de Medicamentos (LCQMed), Departamento de Farmácia, Universidade Federal do Rio Grande do Norte (UFRN), Natal 59010-180, Brazil; ^6^Laboratório de Fitoquímica, Departamento de Botânica, Universidade de São Paulo (USP), São Paulo 05508-090, Brazil

## Abstract

*Commiphora leptophloeos* is widely used in folk medicine without any scientific basis. Considering this, the aim of this study was to evaluate the chemical profile and the antioxidant activity of *C. leptophloeos* leaf extracts using *in vitro* and *in vivo* assays. Six extracts were obtained from fresh leaves using a serial extraction (nonpolar to polar solvents). These extracts were first evaluated with the presence of phytochemical compounds using the methods thin layer chromatography (TLC), ultrahigh performance liquid chromatography (UHPLC-DAD), and high performance liquid chromatography, both with diode array detection (HPLC-DAD). Based on the compounds identified, it was used some bioinformatics tools in order to identify possible pathway and gene targets. After that, the antioxidant capacity from these extracts was analysed by *in vitro* assays and *in vivo* assays using *Caenorhabditis elegans* model. Phytochemical analyses showed the presence of polyphenols, such as rutin, vitexin, and quercetin diglycosides in all extracts, especially in ethanol extract (EE) and methanol extract (EM). Bioinformatics analysis showed these polyphenols linked to antioxidant pathways. Furthermore, EE and EM displayed a high antioxidant capacity in DPPH and superoxide radical scavenging assays. They also had no effect on cell viability for 3T3 nontumour cell. However, for B16-F10 tumour cell lines, these extracts had toxicity effect. *In vivo* assays using *C. elegans* N2 showed that EE was not toxic, and it did not affect its viability nor its development. Besides, EE increased worm survival under oxidative stress and reduced intracellular reactive oxygen species (ROS) levels by 50%. Thus, *C. leptophloeos* EE displayed an important *in vitro* and *in vivo* antioxidant capacity. The EE extract has polyphenols, suggesting that these compounds may be responsible for a myriad of biological activities having this potential to be used in various biotechnological applications.

## 1. Introduction

Medicine plants have a millenary practice in some cultures [1]. It is known that plants produce different intermediate compounds (phenolic compounds, terpens, and alkaloids), and these compounds may have a pharmacology activity like antioxidant role, anticancer agent, and dietary supplementation [2]. According to Newman and Cragg [3] from the period from 1981 to 2014, half of the new molecules approved were originated from medicinal plants. Due to this, medicinal plants have been receiving a special attention in order to identify new bioactive molecules that may be used in medicine for the different diseases such as antioxidant, antimicrobial, antiproliferative, antifungal, antiparasitic, and immunomodulatory [4, 5].

Many age-related and chronic illnesses like diabetes, cardiovascular disease, Parkinson's disease, Alzheimer's disease, inflammatory diseases, and cancer have been associated with oxidative stress due to an imbalance between reactive oxygen species (ROS) production and degradation, consequently increasing the ROS levels [6, 7]. Plant phenolic compounds are known as potent antioxidants, which are crucial for the maintenance of redox homeostasis [4, 6, 7].

*Commiphora leptophloeos* (Mart.) J. B. Gillett. (Burseraceae) is a native species from Caatinga, an exclusive Brazilian biome characterised by low water availability, high temperatures, and high light. Pereira et al. [8] reported on the antimicrobial activity of methanol extract obtained from barks of this plant. However, no data pertaining to its chemical profile and potential antioxidant effects exist. Therefore, the chemical profile of *C. leptophloeos* leaf extracts and the antioxidant activity was investigated in this study, by *in vitro* and *in vivo* assays using antioxidant assays, the 3T3 and B16F10 cell lines as well as the *C. elegans* worms.

## 2. Materials and Methods

### 2.1. Reagents

Potassium ferricianyde (702587), ferrous sulfate II, trichloroacetic acid, Folin-Ciocalteu reagent, sulfuric acid, and Folin-Ciocalteu reagente (109001) were purchased from Merck (Darmstadt, Germany). Nitro blue tetrazolium (NBT), monosaccharides, ethylenediaminetetraacetic acid (EDTA) (E9884), D-glucose (5767), gallic acid (G7384), ascorbic acid (A5960), bovine serum albumin protein (BSA), methionine (M9625), 3-(4,5-dimethylthiazolyl-2)-2,5-diphenyltetrazolium bromide (MTT–M2128), sodium phosphate (S7907), pyrocatechol violet (P7884), riboflavin (R9504), ammonium molybdate, 2,7-dichlorofluorescein (H2DCFDA-D6883), and tert-butyl hydrogen peroxide (t-BOOH-458139) were purchased from Sigma-Aldrich Co. (St. Louis, MO, USA). DPPH (2,2-diphenyl-1-picrylhydrazyl) were purchased from Fluka (Seelze, Germany). Dimethylsulphoxide (DMSO) were purchased from CRQ (São Paulo, Brazil). Sodium bicarbonate, nonessential amino acids, and phosphate-buffered saline (PBS) were purchased from Invitrogen Corporation (Burlington, ON, Canada). Dulbeccos modified Eagles medium (DMEM) and fetal bovine serum (FBS) were obtained from CULTILAB (Campinas, SP, Brazil). Penicillin and streptomycin were obtained from Gibco (Fort Worth, TX, USA). All other solvents and chemicals were of analytical grade from Synth, Diadema, SP, Brazil. The standards used in the HPLC-DAD and UPLC-DAD were 3-hydroxicinnamic acid (501-52-0), benzoic acid (65-85-0), caffeic acid (331-39-5), cinnamic acid (621-82-9), chlorogenic acid (327-97-9), ellagic acid (476-66-4), ferullic acid (1135-24-6), gallic acid (149-91-7), gentisic acid (490-79-9), O-coumaric acid (583-17-5), p-coumaric acid (501-98-4), rosmarinic acid (20283-92-5), sinapic acid (530-59-6), apigenin (520-36-5), apiin–apigenin-7-(2-O-apiosylglucoside) (26544-34-3), kaempferol (520-18-3), kaempferol 3-O-D-galactoside (23627-87-4), 3-O-methylkaempferol (1592-70-7), biorobin–kaempferol 3-O-*β*-robinobioside (17297-56-2), nicotiflorin-kaempferol 3-O-*β*-rutinoside (17650-84-9), catechin (154-23-4), crysin–5,7-dihydroxyflavone (480-40-0), chrysoeriol–3′-O-methylluteolin (491-71-4), daidzein–4,7-dihydroxyisoflavone (486-66-8), epicatechin (490-46-0), galangin (548-83-4), genistein–4′,5,7-trihydroxyisoflavone (446-72-0), gossypetin–8-hydroxyquercetin (489-35-0), hesperidin (520-26-3), hesperitin (520-33-2), hispidulin–6-methoxyapigenin (1447-88-7), homoorientin–luteolin 6-C-*β*-D-glucoside (4261-42-1), isorhamnetin–3′-methoxyquercetin (480-19-3), cacticin–isorhamnetin 3-O-*β*-galactoside (6743-92-6), isorhamnetin 3-O-*β*-D-glucoside (5041-82-7), keioside-isorhamnetin 3-O-robinobioside (107740-46-5), narcissin–isorhamnetin 3-O-*β*-rutinoside (604-80-8), luteolin (491-70-3), myricetin (529-44-2), naringenin (480-41-1), neohesperidin (13241-33-3), orientin–luteolin 8-C-glucoside (28608-75-5), pinocembrin (480-39-7), quercetagetin-7-O-glucoside (548-75-4), quercetin (117-39-5), hyperin-quercetin 3-O-*β*-galactoside (482-36-0), isoquercetrin–quercetin 3-O-*β*-glucoside (482-35-9), quercetin 3-O-*β*-gentiobioside (7431-83-6), quercetin 3-O-robinobioside (52525-35-6), quercitrin–quercetin 3-O-rhamnoside (522-12-3), rhamnetin (90-19-7), rutin–quercetin 3-O-*β*-rutinoside (153-18-4), taxifolin–dihydroquercetin (480-18-2), tiliroside–kaempferol 3-O-(6^″^-O-p-coumaroyl) glucoside (20316-62-5), and vitexin–apigenin 8-C-glucoside (3861-93-4). Other standards correspond to a library made in the Phytochemistry Lab, Department of Botany, University of São Paulo.

### 2.2. Plant Material

*Commiphora leptophloeos* leaves were collected in Sumé, Paraíba, Brazil (7° 40′ 08.6^″^ S 36° 51′ 09.0^″^ W) in July 2016. This specie was identified by Dr. Leonardo de Melo Versieux (voucher #22.353-herbarium at Centro de Biociências from UFRN). This work was conducted under SISBIO #56.509-1.

### 2.3. Leaf Extract Preparation

After harvesting, the fresh leaves were transported to the laboratory where it was washed with water to remove impurities and crushed into small pieces with the aid of scissors. *Commiphora leptophloeos* leaves (100 g) were subjected to a serial extraction with hexane (EH), chloroform (EC), ethanol (EE), methanol (EM), and water (EAR-residual aqueous), with solvent proportion of 1 : 10 (*w*/*v*) (100 g fresh leaves to 1000 mL of solvent) for 24 h at 23°C, 150 rpm and under light protection. Another aqueous extract (EA) was prepared according to description of traditional use (100 g fresh leaves to 1000 mL of distilled water). All extracts were filtered on Whatman No. 1 filter paper and dried using a rotary evaporator (TE210, Tecnal, Piracicaba, Brazil) at 40°C. Dried extracts were suspended in DMSO and then diluted with water to a final concentration of 10 mg/mL. Samples were stored at -20°C until use.

### 2.4. Total Phenolic Compounds

The total phenolic compounds were determined using the Folin-Ciocalteau colorimetric method (*λ* = 765 nm) using a spectrophotometer (Biotek Epoch Microplate, California, CA, USA). Each extract (20 *μ*L) was added to a different tube containing 1.580 *μ*L of distilled water, mixed, and then it was added 100 *μ*L Folin-Ciocalteu reagent. The mixture was kept at room temperature for 10 min., then it was added 50 *μ*L of 5% Na_2_CO_3_, and it was mix in a vortex. Samples were kept for 10 min at room temperature and then transferred to water bath at 37°C for 20 min. After this period, samples were read at 765 nm using a spectrophotometer (Biotek Epoch Microplate, California, CA, USA). Gallic acid (Sigma-Aldrich, Saint Louis, MO, USA) was used as a standard, and values were given per g of extract according to Athukorala et al. [9].

### 2.5. Thin Layer Chromatography (TLC)

All samples were subjected to the following: three mobile phases using glass plates with silica gel *F*_254_ (Merck, Germany): (1) ethyl acetate : formic acid : methanol : water (10 : 0.5 : 0.6 : 0.2 *v*/*v*/*v*/*v*); (2) toluene : methanol : water (9 : 1 : 0.1 *v*/*v*/*v*/*v*); (3) ethyl acetate : formic acid : water (8 : 1 : 1 *v*/*v*/*v*). It was used 0.1 mL from each extract to have a quality TLC. The development reagents used were sulphuric vanillin (0.5 g vanillin, 2 mL H_2_SO_4_, 80 mL MeOH), ferric chloride (1 g FeCl_3_, 100 mL MeOH), natural reagent A (0.2 g Difenilborato, 100 mL MeOH), and Dragendorff's reagent (A solution: 0.85 g bismuth subnitrate, 10 mL CH_3_COOH, 40 mL distilled water; solução B: 16 g de KI, 40 mL distilled water). Chromatoplates were visualised under UV light at 365 nm. The colour and the retention factor (*rf*) from the spots were compared to standards (coumarin, luteolin, ellagic acid, quercetin, kaempferol, catechin, isoorientin, caffeic acid, chlorogenic acid, ursolic acid, and gallic acid) (Sigma-Aldrich, Saint Louis, MO, USA) [10].

### 2.6. Ultrahigh Performance Liquid Chromatography with Diode Array Detector (UHPLC-DAD)

Aliquots of 2 *μ*L of the six extracts (EH, EC, EE, EM, EAR, and EA) were filtered using a 0.22 *μ*m membrane (Merck) and was injected in UHPLC (Shimadzu/Prominence UFLC-XR®) equipped with a binary analytical pump (LC-20ADXR), automatic injector (SIL-20ACXR), a photodiode array detector (SPD-M20A), and a system controlled by LC Solution® software. The column used was Poroshell 120 EC-C18 (50 mm × 4.6 mm i.d., 2.7 *μ*m). The mobile phase (flow 0.5 mL/min) was a gradient of 0.1% formic acid (A) and acetonitrile: 0.1% formic acid (B). The following gradient (35 min as the total time of analysis) was applied: 0 min (99% A and 1% B), 3 min (91% A and 9% B), 19 min (52% A and 48% B), 23 min (5% A and 95% B), 24 min (99% A and 1% B), and 30 min (99% A and 1% B). The phenolic compounds were identified in the UV-visible (360 nm), and the retention time was compared with the standards: rutin, quercetin, pyrogallol, kaempferol, and gallic acid (Sigma-Aldrich, Saint Louis, MO, USA).

### 2.7. Ethanol Extract Analysis Using *HPLC-DAD*

According to our biological results, the ethanol extract (EE) profile was determined using the Agilent 1260 chromatograph with DAD detector used. Phenylpropanoid analysis was performed using a C18 column (4.6 mm × 150 mm) with 3.5 *μ*m diameter particle (Zorbax Eclipse Plus C18). The extract was diluted with methanol to a concentration of 1 mg/mL. The injection volume was 3 *μ*L. The flow rate used in the column was 1 mL/min. The EE was analysed at a concentration of 1 mg/mL. The mobile phase used was an elution gradient containing 0.1% acetic acid (A) and acetonitrile (B), and the gradient composition was 10% B (0-6 min), 10-15% B (6-7 min), 15% B (7-22 min), 15-50% B (22-32 min), 50-100% B (32-42 min), and 100% B (42-50 min). The column temperature was 45°C, and detection was done at 352 nm. The identification of phenolic compounds was obtained by comparison of retention time and UV-visible absorption spectra with standards of this substance.

### 2.8. Bioinformatics Analysis

Based on the possible bioactive molecules identified by UHPLC-DAD and HPLC-DAD analysis—rutin, vitexin, and quercetin diglycosides—the Traditional Chinese Medicine System Pharmacology (TCMSP) database was used [11]. The data obtained at TCMSP were used at Kyoto Encyclopedia Genes and Genome (KEGG database) [12]. Then, the pathways were listed in descending order according to *p* value. These data were used to build a network using String 10 version 11 (https://string-db.org/).

### 2.9. In Vitro Antioxidant Activity

#### 2.9.1. Reducing Power Assay

The reducing power of *C. leptophloeos* extracts was quantified by the reduction of potassium ferricyanide into potassium ferrocyanide [9, 13]. The reaction mixture (4 mL) containing the plant extracts at 50, 100, and 250 *μ*g/mL in phosphate buffer (0.2 M, pH 6.6) was incubated with potassium ferricyanide (1% *w*/*v*) at 50°C for 20 min. The reaction was terminated by the addition of 10% trichloroacetic (TCA) solution, distilled water, and 0.1% ferric chloride. The absorbance was measured at 700 nm, using phosphate buffer as a blank. Results were expressed as activity percentage reported by 100 *μ*g/mL of ascorbic acid (standard-Sigma-Aldrich, Saint Louis, MO, USA) according to Athukorala, et al. [9] and Wang et al. [13].

#### 2.9.2. DPPH Radical Sequestration Assay

The DPPH assay was performed according to the method by Shimada et al. [14], using a 96-well plate. Aliquots of extracts (1, 2, and 5 *μ*L) at final concentration of 50, 100, and 250 *μ*g/mL were added, and 150 *μ*M DPPH were added into each well, mixed, and the plate kept at room temperature for 30 min. The absorbance was measured at 517 nm. The DPPH free-radical scavenging activity was calculated as follows:

DPPH scavenging activity : (%) = [1–(A1/A0)] × 100, where A0 is the absorbance of the control (DPPH) and A1 is the absorbance of the samples.

#### 2.9.3. Superoxide Radical Scavenging Activity Assay

This assay was based on the method by Dasgupta and De [15] inside a dissipating chamber of light. This assay was based on the extracts to inhibit the reduction of nitroblue tetrazolium (NBT) in the riboflavin-light-NBT system. The extracts at different final concentrations (100, 250, and 500 *μ*g/mL) were mixed to 3 mL reaction mixture (50 mM phosphate buffer (pH 7.8), 13 mM methionine, 2 mM riboflavin, 100 mM EDTA, and 75 mM NBT) and exposed to a fluorescent lamp for 10 min. Thereafter, absorption was measured at 560 nm—EDTA was used as a control and distilled water as the blank. Results were expressed as percent of radical scavenging:

Percentage of radical scavenging = ([Acontrol − Asample]/[Acontrol − Ablank]) × 100.Acontrol: absorbance of the control; Asample: absorbance of the sample; Ablank: absorbance of the blank.

### 2.10. Cell Viability Assay

Cell viability of the murine fibroblast cell line NIH/3T3 (ATCC CRL-1658TM, Manassas, VA, USA) and B16-F10 cell lines (*ATCC* CRL-6475, Manassas, VA, USA) was evaluated against ethanol extract (EE). The EE was chosen due to the results obtained with *in vitro* antioxidant assays. Cells were grown in Dulbecco's modified Eagle's medium (DMEM) (Cultilab, São Paulo, Brazil) in 75 cm^2^ flasks, supplemented with fetal bovine serum (FBS) (LGC Biotecnologia, São Paulo, Brazil). Cells were seeded into 96-well plates at a density of 5 × 10^3^ cells/well and allowed to attach overnight in 200 *μ*L of medium with 10% FBS (LGC Biotecnologia, São Paulo, Brazil) at 37°C, in 5% CO_2_. After 24 h, the EE was added in three different concentrations: 50, 100, and 250 *μ*g/mL for a period of 24 h at 37°C and 5% CO_2_. Thereafter, the medium and extract were removed and 100 *μ*L of fresh medium and 10 *μ*L of 12 mM MTT (3-(4,5-dimethylthiazol-2-yl)-2,5-diphenyltetrazolium bromide) (Sigma-Aldrich, Saint Louis, MO, USA) dissolved in PBS were added. The plate was incubated for 4 h at 37°C in 5% CO_2_. In order to solubilise the reduced MTT product, 100 *μ*L of absolute ethyl alcohol was added to each well and mixed [16]. The absorbance was measured at 570 nm. The percentage of cell viability was calculated as:

(1) $$\begin{array}{c} \text{Percentage of MTT reduction}=\left(\frac{\text{Abs of sample}}{\text{Abs of control}}\right)\times 100. \end{array}$$

### 2.11. *Caenorhabditis elegans* Strains and Cultivation

The *Caenorhabditis elegans* strains used in this study were the N2 (wild-type) and transgenic line CF1553 (muIs84[pAD76(sod-3::GFP) + rol-6(su1006)]. *Caenorhabditis elegans* were cultivated in Nematode Growth Medium (NGM) with *Escherichia coli* OP50 and maintained at 20°C [17]. Synchronised first larval stage (L1) worms were obtained by treating gravid hermaphrodites with 2% sodium hypochlorite and allowing the eggs to hatch on M9 buffer overnight. For treatment, EE was filtered using a 0.22 *μ*m filter (Merck), and subsequently, it was added to the NGM at a final concentration of either 1 mg/mL or 10 mg/mL. Worms were incubated with *E. coli* OP50 for 48 h.

### 2.12. Effect of Ethanol Extract on *Caenorhabditis elegans* Development

The effects of EE on egg hatching was determined. *Caenorhabditis elegans* eggs were incubated in plates containing NGM and *E. coli* OP50 in the absence or presence of EE at 100, 250, 500, and 1,000 *μ*g/mL. Approximately 50 eggs per plate were added to a total of five plates per treatment. After the incubation period, the number of hatched eggs was analysed using the following formula:

(2) $$\begin{array}{c} \text{Hatching percentage}=\left(\frac{\left(\text{No}.\text{of larvae}\right)}{\left(\text{No}.\text{of eggs}\right)}\times 100\right). \end{array}$$

The other aspect analysed was the body length. The L1 synchronised worms were kept at NGM medium plus *E. coli* OP50 in the absence or presence of EE at 100, 250, 500, and 1000 *μ*g/mL. These plates were kept at 20°C for 48 h. Approximately 25 animals were used per treatment, and each experiment was performed in triplicate. The animals were photographed, and their body length was measured using ImageJ software, version 1.8.

### 2.13. Quantification of Intracellular ROS

Wild-type animals synchronised at L1 were kept on NGM plates with *E. coli* OP50. These worms were treated with EE at concentrations of 0, 100, 500, and 1000 *μ*g/mL for 48 h. Thereafter, worms were washed thrice with M9 buffer and incubated with 500 *μ*L M9 buffer. Afterward, approximately 40 animals were transferred to 96-well plates containing 2 mM H2DCF-DA (2,7-dichlorofluorescein diacetate fluorescent probe), to measure ROS levels. Samples were read using a multilabel microplate reader GloMax®-Multi Detection System (Promega, Wisconsin, USA), with excitation at 490 nm and emission at 510-570 nm, and the mean values were calculated. Readings were done intermittently every 30 min during 4 h. This assay was performed in triplicate.

### 2.14. Survival Test under Oxidative Stress Conditions

Synchronised wild-type animal at L4 were treated with EE at concentrations of 0, 100, 500, and 1000 *μ*g/mL for 48 h. Thereafter, approximately 50 animals were transferred to 24-well plates with NGM containing 10 mM t-BOOH (tert-butyl hydroperoxide). Survival was monitored every three hours until all animals were considered dead (no movement). Each group was made up of 10 animals, and each assay was analysed five times [18].

### 2.15. Analysis of sod-3::GFP Expression

The transgenic line CF1553 worms were treated with EE at 0, 100, 500, and 1000 *μ*g/mL for 48 h. Thereafter, 25 worms were transferred to microscopic slides with 1% agarose and covered. These worms were photographed using an Olympus BX51 fluorescence microscope using a 10-fold eye magnification and 365 nm excitation filter. The GFP fluorescence intensity was analysed using ImageJ Software version 1.8 (https://imagej.net).

### 2.16. Statistical Analysis

The results were expressed as the mean ± standard deviations. Each assay was repeated 2–3 times and was performed in triplicate or quintuplet. Statistical analysis was performed using GraphPad Prism 6.0 (2014), and the results were obtained using one-way analysis of variance (ANOVA) followed by Tukey's post hoc analysis (*P* < 0.05).

## 3. Results

### 3.1. *Commiphora leptophloeos* Leaf Extracts Are Rich in Phenolic Compounds

In this study, a serial approach using solvents from different polarity (nonpolar to polar) was done in order to obtain five extracts from *C. leptophloeos* leaves: hexane (EH), chloroform (EC), ethanol (EE), methanol (EM), and water (EAR-residual aqueous), and the sixth extract was prepared only with water-EA. The total phenolic content of compounds obtained ranged from 5.44 ± 0.4 mg/g to 126.41 ± 1.8 mg/g. The EH had 10.18 ± 0.4 mg/g, while EC had 42.47 ± 0.2 mg/g, EAR had 24.27 ± 0.6 mg/g, and EA had 5.44 ± 0.4 mg/g. The highest values were obtained from the EM (126.41 ± 1.8 mg/g) and EE (105.67 ± 1.7 mg/g).

**Figure 1:**
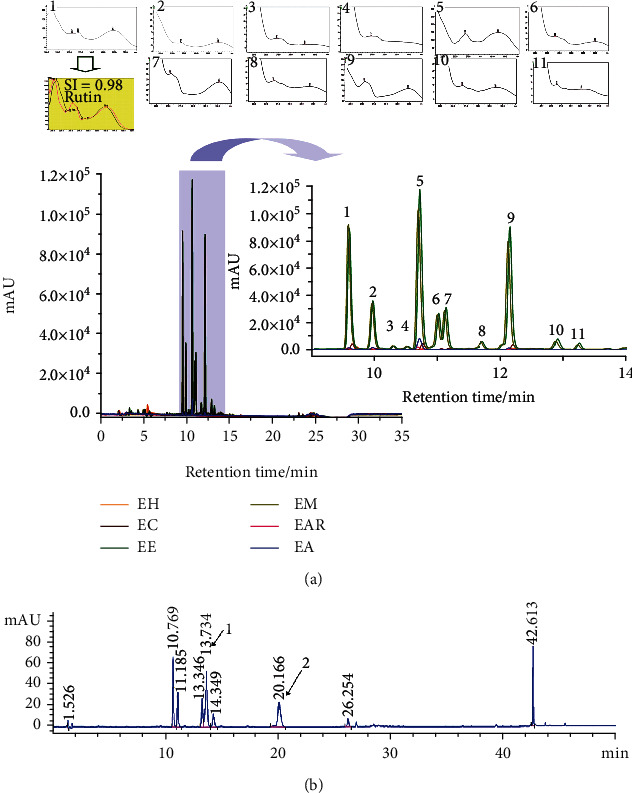
UHPLC chromatogram. The extracts: EH (hexane extracT), EC (chloroform extract, EE (ethanol extract), EM (methanol extract), EAR (residual aqueous extract), EA (aqueous extract) were analysed using UHPLC-DAD at 360 nm. In (a), the chromatogram obtained for each extract is shown. Green represents the chromatogram detail obtained for the EE. Peak number 1 corresponds to rutin. The *x*-axis corresponds to the retention time (min) of each peak. The *y*-axis corresponds to the intensity of the peaks in mAU. In (b), the EE analysis using HPLC-DAD was shown. The detection wavelength was set at 360 nm, the *x*-axis corresponds to the retention times (min) of each peak, and the *y*-axis corresponds to the intensity of the peaks in mAU. The peak absorption spectra #1 corresponds to vitexin #2 to quercetin diglycosides.

The presence of terpenes was observed in TLC analysis for the EH. The presence of terpenes, phenolic acids, and flavonoids was detected in the EC; terpenes, phenolic compounds, flavonoids, saponins, and tannins were observed in both the EE and EM. Phenolic compounds were detected for EAR and not observed for EA. Moreover, the fingerprinting analysis of extracts via UHPLC-DAD allowed the detection of several compounds similar to phenols at higher concentrations mainly in EE and EM according to UV spectra (see Figure 1(a)). In green represented the EE, and the peak #1 may correspond to the rutin flavonoid (tr = 9.6). Regrettably, the other peaks in Figure 1(a) may not be determined by our analysis.

Based on the biological results obtained with EE, then it was analysed using HPLC-DAD at 360 nm. The presence of vitexin—peak 1 (tr = 13,734); peak 2), and peak 2 suggested the presence of quercetin diglycosides (tr = 20,166) (see Figure 1(b)).

### 3.2. Bioinformatics Analysis

**Figure 2:**
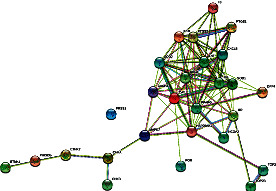
Gene network from *Commiphora leptophloeos* bioactive molecules. The data obtained from KEEG were used to build a network using SRTING10 version 11. Each circle corresponds to a gene; the lines are how these genes are connected between each other. The blue line corresponds to curate database; pink determined experimentally; yellow data from text mining; black coexpression; green colour means gene neighbourhood.

Bioinformatics may be a powerful tool to make a data mining from potential targets using the bioactive molecules presented in the extracts. The pharmacological analysis was done in order to identify the potential targets from the three bioactive molecules presens at *C. leptophloeos* leaf extract: rutin, vitexin, and quercetin diglycosides. It may be observed that these molecules may be associated to pathways that were related to anti-inflammatory, cardiovascular disease, neurodegenerative disease, cancer, and others. It was observed more than 56 targets (see Table 1). Moreover, when the overlap was analysed, then some gene argets were PTGS2, PIK3CG, CASP3, MAPK7, SOD, CAT, ETNK1, ENTK2, HSP90, TNF, IL1*β*, and IL6 (see Table 1, Figure 2) Furthermore, the bioinformatics approach proposed that the bioactive molecules identified at *C. leptophloeos* may have an antioxidant activity.

In Figure 2, it may be observed how the genes identified at Table 1 may interact. Although many of these genes are correlated to the immune system, there is also a correlation to oxidative stress as the SOD2 (Sodium Dismutase) and CAT (Catalase) was observed for example. Then, this approach showed that it was important to analyse if these extracts (complex mixture of bioactive molecules) may have antioxidant activities and others activities.

### 3.3. In Vitro Antioxidant Assays and In Vivo Cell Viability

**Figure 3:**
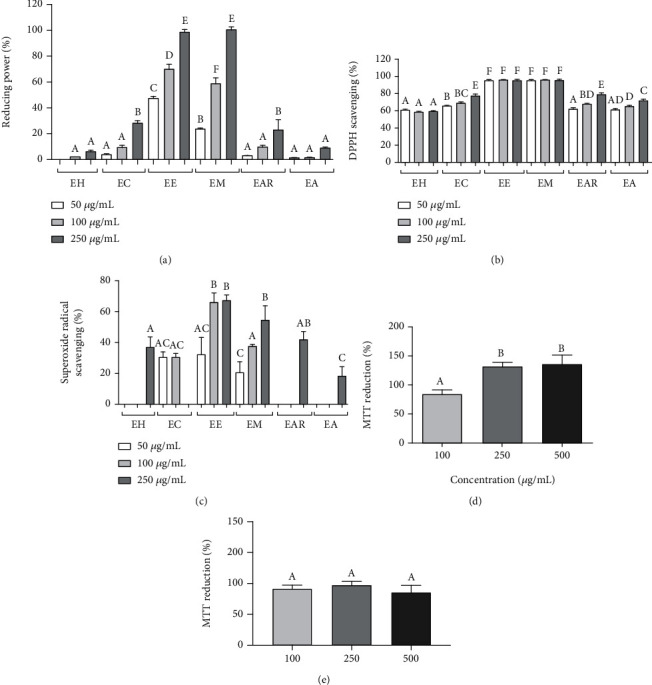
Antioxidant activity assays and cell viability assay. The antioxidant assays were performed using the *Commiphora leptophloeos* extract concentration at 50, 100, and 250 *μ*g/mL. (a) Reducing power assay. The *x*-axis corresponds to the different extracts and concentrations used. The *y*-axis corresponds to the activity percentage, based on the standard curve using ascorbic acid as a standard. (b) DPPH assay. The *x*-axis corresponds to the different extracts and concentration. The *y*-axis corresponds to the DPPH percentage scavenging. (c) Superoxide radical scavenging assay. The *x*-axis corresponds to the different *C. leptophloeos* extracts. The *y*-axis corresponds to the percentage scavenging. (d) Cell viability of the NHI/3T3 cell line after 24 h of ethanol extract (EE) treatment. The *x*-axis corresponds to EE concentration at 100 *μ*g/mL, 250 *μ*g/mL, and 500 *μ*g/mL. The *y*-axis corresponds to the percentage of cell viability via MTT reduction. (e) Cell viability of the B16F10 tumour cell line after 24 h of EE treatment. The *x*-axis corresponds to EE concentration at 100 *μ*g/mL, 250 *μ*g/mL, and 500 *μ*g/mL. The *y*-axis corresponds to the cell viability percentage via MTT reduction. EH: hexane extract; EC: chloroform extract; EE: ethanol extract; EM: methanol extract; EAR: residual aqueous extract; EA: aqueous extract. All assays (a–e) were done in triplicate for each extract, and the data obtained were analysed using ANOVA and Tukey's test (*P* ≤ 0.05). Different letters (a, b, c, d, e, and f) correspond to significant differences.

Considering the presence of phenolic compounds in the order EM > EE > CE > EAR > EH > EA, the *in vitro* antioxidant capacity of these extracts were then evaluated using seven different assays. The extract concentrations that were analysed were 50, 100, and 250 *μ*g/mL. The assays that had activity were presented. The reducing power potential increased in a dose-dependent manner (see Figure 3(a)). A 100% reducing power potential was observed at 250 *μ*g/mL for both EE and EM (see Figure 3(a)). The DPPH assay displayed saturation as the value reached 100% of DPPH scavenging at the lowest concentration (50 *μ*g/mL) (see Figure 3(b)). Moreover, the EE and EM exhibited the highest superoxide radical scavenging potential (see Figure 3(c)). The EE reached 65% at 100 *μ*g/mL.

Since EE presented the highest antioxidant activity amongst all extracts, its effect on NIH/3T3 cell viability was analysed (see Figure 3(d)). As expected, no effect on cell viability was observed. Furthermore, higher concentrations of EE seemed to increase cell metabolism and viability when measured by the MTT assay (see Figure 3(d)). On the other hand, when it was evaluated, the EE effect on B16F10 tumour cell line was observed a cytotoxicity effect as it may be observed a reduction on cell viability in a range of 30% at 100 *μ*g/mL up to 45% at 500 *μ*g/mL extract concentration.

### 3.4. Effect of *Commiphora leptophloeos* EE on *Caenorhabditis elegans* Development

**Figure 4:**
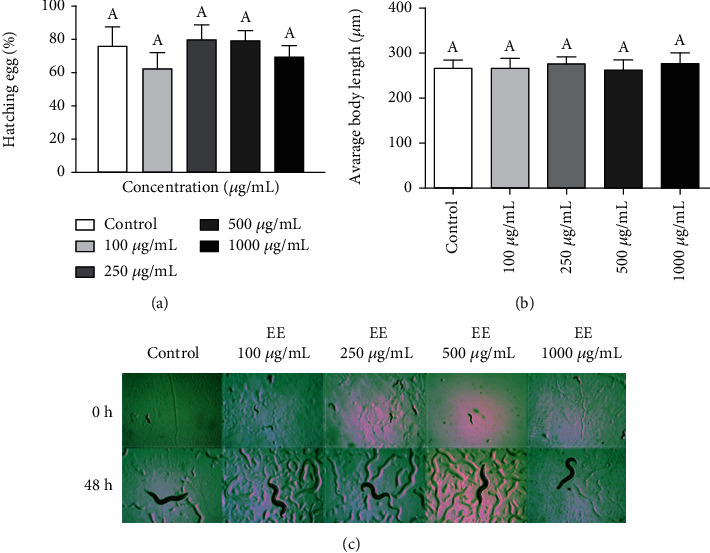
Ethanol extract (EE) effect on *Caenorhabditis elegans* wild-type growth and development. Wild-type *C. elegans* animals were treated with EE at 0, 100, 250, 500, and 1000 *μ*g/mL concentration. (a) Egg hatching assay. The *x*-axis corresponds to concentrations of EE. The *y*-axis corresponds to the percentage of egg hatching. (b) Body length was measured using Image J software. The *x*-axis corresponds to EE concentrations used. The *y*-axis corresponds to body length. (c) Image from the animal at 0 h and 48 h of EE treatment at the different concentrations. Three independent experiments were performed, with three replicates per treatment (A-B-C). The data were analysed by ANOVA and Tukey's test (*P* ≤ 0.05).

The nematode *C. elegans* was used to analyse the EE effect on animal development as well as its antioxidant capacity using this *in vivo* model. The EE concentration used were 0, 100, 250, 500, and 1000 *μ*g/mL. The 1000 *μ*g/mL concentration was used due to the *C. elegans* cuticle. The effect of EE on the development *C. elegans* was evaluated, and any effects on egg hatching at any of the concentrations analysed were not observed (see Figure 4(a)). Moreover, any modifications in body length (see Figure 4(b)) nor in animal development (see Figure 4(c)) were not observed.

### 3.5. In Vivo Antioxidant Effect of EEs Using *Caenorhabditis elegans*

**Figure 5:**
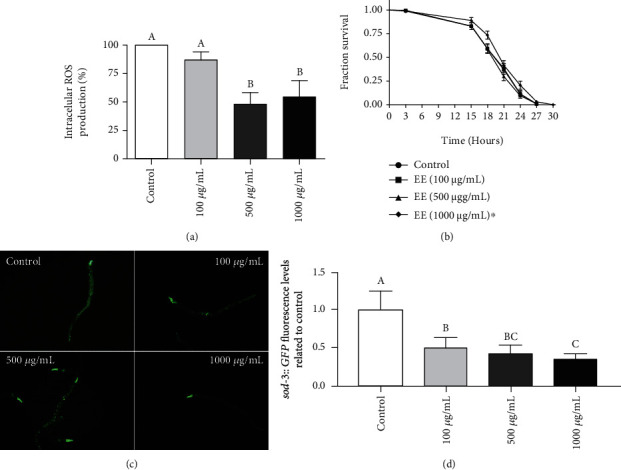
Ethanol extract (EE) antioxidant effect on *Caenorhabditis elegans* model. (a) Levels of reactive oxygen species (ROS) in N2 wild-type animals treated with the *C. leptophloeos* EE for 48 h. (b) Survival assay of N2 wild-type animals under oxidative stress conditions with t-BOOH. Animals were treated with 0, 100, 500, or 1000 *μ*g/mL of EE at L1 to L4. The *x*-axis corresponds to the hours observed (3 to 30 h), and the *y*-axis, to the survival fraction. Three independent experiments were performed, with three replicates per treatment. The EE treatment was compared to the control by the Log-rank test (*P* < 0.0001). (c) *sod-3::GFP* expression in *C. elegans* after EE treatment 0, 100, 500, or 1000 *μ*g/mL. The images were taken using the Olympus BX51 microscope. (d) *sod-3::GFP* expression was quantified. The *x*-axis corresponds to different concentrations of *C. leptophloeos* EE. The *y*-axis corresponds to GFP expression quantification. For all assays, three independent experiments were performed, with three replicates per treatment. Different letters correspond to significant differences. Statistical analysis was performed using ANOVA and Tukey test (*P* ≤ 0.05).

Since EE exhibited an *in vitro* antioxidant capacity and had any cytotoxic effects on cell viability and on *C. elegans* development, it was essential to evaluate the effect of EE treatment on ROS production using the *C. elegans* model. The EE treatment at 500 and 1000 *μ*g/mL was able to reduce approximately 50% of the intracellular ROS level when compared to the untreated control (see Figure 5(a)). Furthermore, it was also observed that the EE concentration of 1000 *μ*g/mL increased survival under oxidative stress induced by t-BOOH (see Figure 5(b)). The mean survival time of 1000 *μ*g/mL of EE-treated animals increased by 5% compared to the untreated control. Moreover, it was observed that the expression level of *sod-3::GFP* transgenic animals was reduced by approximately 50% on GFP expression, when treated with any EE concentration (see Figures 5(c) and 5(d)).

## 4. Discussion

Over the years, medicinal plants have received growing attention in research for the identification and characterization of new intermediate metabolites that may have a different biological effect [4]. Illnesses like cancer, diabetes, cardiovascular disease, inflammatory disease, aging, Parkinson's disease, and Alzheimer's disease have been associated with oxidative stress. The antioxidant effect of plant intermediate metabolites have an important impact on the maintenance of cellular redox homeostasis and could potentially influence the mechanism of these diseases [5, 19–23].

*Commiphora leptophloeos* is used in folk medicine for gastritis, anti-inflammatory, asthma, and bronchitis [24, 25] but no scientific information. The data in this study showed that all the extracts displayed antioxidant activity at in the DPPH assay. Considering that the antioxidant activity has three steps: initiation, propagation, and termination [26–28]; our data showed that the EE and EM had activity at the initiation (DPPH and reducing power) and termination phase (superoxide radical scavenging).

Pereira et al. [8] worked with *Commiphora leptophloeos* stem bark, and they also identified the presence of phenolic compounds, flavonoids, and sugars. Our TLC, UHPLC, and HPLC data showed the presence of phenolic compounds such as rutin, vitexin, and possible quercetin diglycosides, especially in EE *C. leptophloeos*. The bioinformatics analysis associated to the phenolic compounds identified possible gene targets that were associated to antioxidant and anti-inflammatory pathways. It has been observed that misbalance between ROS production and degradation may produce an oxidative stress, which may be associated to inflammatory process as well as to different diseases [19]. Moreover, it has been shown that phenolic compounds as rutin, quercetin, and others may acts in signalling pathways involved in the inflammatory process [19]. Soukhtanloo et al. [22] showed that NF-kb signalling pathway may be a target for some natural products. Choudhari et al. [5] also showed the use of phytochemicals were promising at preclinical treatments. Moreover, He et al. [29] showed that vitexin and isovitexin, active phytochemicals in medicinal plants, have a broad spectrum effect including antioxidant, antiproliferative, anti-inflammatory, and antihyperalgesic effects [29, 30]. Furthermore, a neuroprotective effect that increases viability and impacts LDH release was observed. This could potentially influence Alzheimer's disease due to its neurological protection. The results showed the presence of rutin and probably the presence of quercetin diglycosides in EE *C. leptophloeos* extracts. These flavonoids showed promising antioxidant, anti-inflammatory, and immunoprotective activities and may be used to treat cardiovascular diseases, diabetes, asthma, and cancer [6, 31, 32]. The other flavonoid identified in *C. eptophloeos* extracts was rutin. It is commonly found in dark-coloured fruits that are known to have antioxidant, anti-inflammatory, neuroprotective, and other biological activities [33]. Furthermore, the chemical composition of *C. leptophloeos* is similar to other species from the genus [34, 35].

The evaluation of cell toxicity is an important step in the discovery and application of new drugs. Thus, the data of cell lines and the *C. elegans* model showed that the EE had no *in vivo* cytotoxic effects on the NIH/3T3 cell line, only for B16F10 tumour cell line. Moreover, EE did not affect *C. elegans* egg hatching, body length, and development, which are all important parameters for toxicity [36, 37]. Literature, it showed that the flavonoids identified did not affect cell viability. However, as plant extracts contain a mixture of phytocompounds, it was important to analyse this aspect using cell lines and the *C. elegans* model [4, 38, 39].

Furthermore, *C. elegans* data reinforce the outcome of the bioinformatics and *in vitro* antioxidant assays as it verified that EE have the potential to reduce ROS production, even when animals were treated with t-BOOH (oxidative agent). Moreover, during oxidative stress, excessive production of ROS may promote lesions on DNA as well as promote cell damage. The compounds present in extracts reduced or neutralised ROS effects; thus, they are important for redox homeostasis [29, 32]. Kampkotter et al. [40] showed that treatment with 100 *μ*M of rutin reduced ROS levels by 15% in *C. elegans.* The data presented here also showed an effect on life span after EE treatment in animals that have been associated with the antioxidant capacity.

In this study, a reduction of *sod-3::GFP* expression (reporter gene) was observed in animals treated with EE. This reduction may be associated with the ROS scavenging effect of EE. Similar results were also observed when animals were treated with quercetin or *Anacardium occidentale* extracts [41, 42]. The data suggest that these extracts may act synergistically with phenolic compounds to protect cells and *C. elegans* from oxidative stress by assisting in ROS reduction, consequently helping to maintain cellular homeostasis [28, 38, 39, 42–44].

## 5. Conclusions

The results presented here showed the antioxidant capacity of *C*. *leptophloeos* leaf extracts, especially for ethanol extract (EE). Our data showed the presence of phenolic compounds (rutin, vitexin, and quercetin diglycosides, amongst others), and no cytotoxic effects were observed on cell viability and *C. elegans*. Furthermore, potential antioxidant activity was observed using bioinformatics, *in vitro* and *in vivo* assays. The intermediate metabolites observed in ethanol extract (EE) may potentially reduce ROS effects and consequently maintain redox homeostasis. Thus, *Commiphora leptophloeos* is a promising plant species for future studies on biological activity as oxidative stress is involved in different chronic diseases.

## Figures and Tables

**Table 1:** Data obtained from KEGG using the rutin, vitexin, and quercetin diglycosides as target after the overlap analysis.

KEGG Brite (*Homo sapiens*)	Adjusted *p* value	Corrected *p* value	Related genes (targets)
Immune system	6.61*e*-19	1.22*e*-16	PTGS2; CASP3; CXCL8; IL6; TNF; MAPK7; HSP90AA1; IL1B; RELA
Cardiovascular disease	7.83*e*-09	7.62*e*-08	RELA; HSP90AA1; IL1B; TNF; MAPK7; IL6; CXCL8;
Infectious disease bacterial	2.27*e*-12	1.05*e*-10	CASP3; CXCL8; IL6; TNF; IL1B; RELA
Infectious disease viral	4.03*e*-10	6.77*e*-09	PRSS1; CASP3; CXCL8; IL6; TNF; IL1B; RELA; PTGS2; PIK3CG
Infectious disease parasitic	1.83*e*-09	2.37*e*-08	IL6; CXCL8; IL1B; TNF; RELA
Neurodegenerative disease	8.18*e*-07	4.32*e*-06	IL6; IL1B; SOD1; TNF; CASP3
Pathways in cancer	5.16*e*-09	5.30*e*-08	PTGS2; CASP3; CXCL8; IL6; PPARG; HSP90AA1; RELA
Nervous system	2.52*e*-05	8.48*e*-05	PTGS2; PTGS1; CASP3
Metabolic pathways	1.42*e*-08	1.19*e*-07	PTGS2; PTGS1; PIK3CG; AR; ETNK2; ETNK1; CAT; CHKA; CHKB
Lipid metabolism	1.65*e*-07	1.05*e*-06	ETNK1; CHKA; CHKB; ETNK2
Development and regeneration	4.8*e*-07	2.71*e*-06	PPARG; IL1B; TNF; RELA
Cell growth and death	4.11*e*-05	0.000135	RELA; TNF; CASP3; IL6; CXCL8; RELA; HSP90AA1; IL1B
Transcription misregulation in cancer	2.07*e*-06	1.00*e*-05	IL6; CXCL8; RELA; PPARG
Cancer	1.36*e*-05	5.24*e*-05	PTGS2; RELA; CASP3

## Data Availability

The data obtained in this study are available from the corresponding author upon request.
